# Parasuicidal poisoning by intramuscular injection of insecticide: A case report

**DOI:** 10.3892/etm.2013.1216

**Published:** 2013-07-11

**Authors:** HUIMIN LIU, BAOTIAN KAN, XIANGDONG JIAN, WEI ZHANG, QIAN ZHOU, JIERU WANG

**Affiliations:** Department of Poisoning and Occupational Disease, Qilu Hospital of Shandong University, Jinan, Shandong 250012, P.R. China

**Keywords:** organophosphate insecticide, parasuicide, injection, intramuscular

## Abstract

Suicidal poisoning by ingestion of organophosphate (OP) insecticides represents a serious emergency with a high mortality rate. However, attempted suicide via the parenteral route has rarely been reported. The present study reports a case of parasuicide by self-injection of an organophosphorus insecticide (phoxim, phenylglyoxylonitrile oxime O,O-diethyl phosphorothioate) into the distal region of the left arm. The patient developed necrosis at the injection site and an abscess of the affected limb following injection. A fasciotomy and surgical debridement resulted in the symptoms of the patient disappearing within a few days and were vital in shortening the course of the disease.

## Introduction

As society develops, the population is faced with increasing social pressures and family conflicts. Suicide rates are also increasing, particularly among young people, and ingestion of agrochemicals is one of the most common methods used in China (1). In developing countries, household insecticides are easily obtained, contributing to the occurrence of suicide. Household insecticides are classified as chlorinated hydrocarbons, acetylcholinesterase inhibitors and botanical agents. Acetylcholinesterase inhibitors include carbamates and organophosphates (OPs) (2). Organophosphorus cholinesterase-inhibiting insecticides are divided into two groups, highly toxic and moderately toxic. Phoxim falls into the latter group. The use of the parenteral route to self-administer this poison is extremely rare (3,4). Fratello *et al*(3) presented a case of acute OP ester poisoning via a parenteral route. Güven *et al*(4) reported the first case of OP intoxication by intravenous (i.v.) injection. However, to the best of our knowledge, the present study is the first reported case of suicidal poisoning by intramuscular injection of OP in China. The study was approved by the Ethics Committee of Qilu Hospital of Shandong University. (Jinan, China). Written informed consent was obtained from the patient.

## Case report

A 33-year-old married male was admitted to The Department of Poisoning & Occupational Disease, Qilu Hospital of Shandong University, Shandong, China, following attempted suicide by injection of phoxim (~10 ml) into the distal region of the left arm. Upon interview, the patient confirmed that the suicide attempt had occurred 9 days prior to admission. The patient was discovered 3 h after the incident and a ventouse was used at the injection site. No systemic signs of intoxication were observed at the time. However, the patient later developed diarrhea and vomiting, and was admitted to the local hospital for emergency treatment. The patient was immediately diagnosed with organophosphorus insecticide poisoning, and i.v. atropine and pralidoxime therapy was administered (specific quantities unknown). The patient was referred to the Department of Poisoning and Occupational Disease for further treatment nine days later.

**Figure 1:**
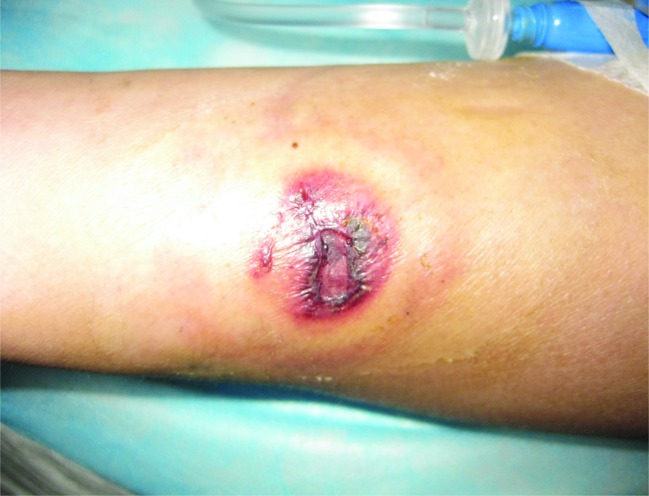
A pale-cicatrix was visible following self-injection of phoxim into the left arm.

The patient had previously experienced a craniocerebral trauma. On physical examination, the patient was conscious, with a blood pressure of 120/75 mmHg, a pulse of 85 beats/min, pupils of equal roundness and size (left, 2 mm; right, 2 mm) and a positive light reflex in both eyes. The patient exhibited spontaneous respiration and his respiratory sounds were exaggerated at a rate of 25 breaths/min. Neurological and abdominal examinations were normal. The distal region of the left arm was swollen and exhibited an erythematous reaction. The diameter of the pale-cicatrix was ~2 cm (Fig. 1). Serum cholinesterase levels were 200 IU/l (normal reference, 5,900–12,220 IU/l). An i.v. infusion of crystalloid solution was administered. Atropine therapy (i.v.; 2 mg twice a day followed by 2 mg every 4 h) was continued for 2 days and an intramuscular injection of pralidoxime chloride (0.5 g) was administered once daily. Atropine therapy was continued for 15 days. This therapeutic regimen was continued for 15 days; however, the doses were changed during this time. It was difficult to titrate the atropine dose as the patient had received i.v. atropine and pralidoxime chloride therapy prior to admission. Furthermore, the dose of atropine was changed a number of times according to serum cholinesterase levels.

**Figure 2:**
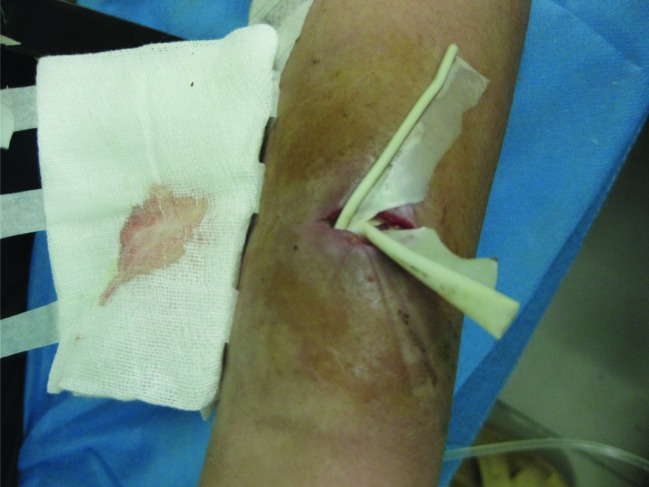
A wound was visible following fasciotomy and surgical debridement of the left arm.

For two days following admission, serum cholinesterase levels were 200 IU/l. On day 4, serum cholinesterase levels were 341 IU/l. Laboratory tests revealed a white blood cell (WBC) count of 14.38×10^9^/l and lactate dehydrogenase (LDH) and LDH1 isoenzyme levels were slightly increased. Cranial computed tomography (CT) revealed a crevice in the frontal bone of the patient, likely caused by the previous craniocerebral trauma, and no abnormalities were observed in the brain parenchyma. A chest CT scan revealed left lower pulmonary fibrotic foci and an abdomen CT scan was normal. On day 5, serum cholinesterase levels were 324 IU/l and the symptoms were not improved. On day 7, the patient deteriorated, becoming drowsy and developing weakness. Laboratory findings were as follows: cholinesterase (CHE), 334 IU/l; K, 3.34 mmol/l; LDH, 236 U/l; WBC, 18.12×10^9^/l; and platelets (PLT), 311×10^9^/l. On day 9, serum cholinesterase levels were 433 IU/l. Following day 12, the patient appeared to improve, exhibiting alleviated symptoms, including reduced pain and drowsiness, and an increased serum cholinesterase level of 1240 IU/l. On day 14, the patient accidentally scratched the cicatrix on the upper left limb. This led to bursting of the skin, pouring bloody liquid, which had a heavy OP insecticide odor. Immediate surgical debridement, incision and drainage were carried out. The necrotic tissue at the injection site was excised and the dressing was changed regularly (Fig. 2). On day 15 after admission, serum cholinesterase levels were elevated to 1,877 IU/l, although this remained below the normal range. Routine blood, kidney and liver function examinations revealed normal values. Atropine and pralidoxime chloride were discontinued. By day 21, serum cholinesterase levels had increased from 1,877 to 3,823 IU/l and symptoms had resolved completely. The patient was discharged from the hospital.

## Discussion

Phoxim is an OP compound. OP insecticides strongly inhibit cholinesterase and pseudocholinesterase activities (5). Phoxim is also known as Volaton or Baytion (phenylglyoxylonitrile oxime O,O-diethyl phosphorothioate) and is a highly effective OP pesticide with a low toxicity. It is used as an insecticide and acaricide, mainly to treat grains, including rice, sorghum, wheat, corn and beans, prior to storage or before sowing to prevent attack by worms. OP poisoning occurs mainly via the gastrointestinal and respiratory tracts and the skin. Exposure to the chemical via other routes causes varying symptoms. The type and severity of symptoms depend on the amount of OP involved and the nature of the exposure. The initial treatment of poisoning focuses on ensuring adequate oxygenation, followed by the administration of atropine to antagonize the muscarinic and central nervous system effects of the OP (6). Pralidoxime is usually used in the case of respiratory depression, muscle fasciculations or muscular weakness to antagonize the toxicity of OPs on nicotinic synapses (7). The dose of atropine and pralidoxime should be controlled flexibly. In the present case, signs or symptoms of systemic toxicity resulting from the intramuscular injection of OP did not manifest immediately. This may have been due to the amount of OP injected and the low level of OP transition to the circulatory system (8). Fasciotomy and surgical debridement were not performed until the patient accidentally scratched the cicatrix. This case differs from previous studies (8–10), as the cicatrix was old and scabbed upon referral, thus it was not considered to be significant. Measurements of plasma cholinesterase and erythrocyte cholinesterase activities are the most useful methods for confirming OP exposure. Prior to the fasciotomy and surgical debridement, serum cholinesterase levels fluctuated between 200 and 433 IU/l; however, these were rapidly elevated following surgery. Therefore, we suggest that immediate application of fasciotomy and surgical debridement should be established to prevent later complications, including ischemic contracture or limb amputations, which have been shown to occur in a previous study (11). In the present case, a prolonged course of treatment was required since fasciotomy and surgical debridement were not performed immediately. Therefore, immediate fasciotomy and surgical debridement are indicated to be necessary. Furthermore, surgical debridement may also aid in the clearance of toxic substances and contribute to the prevention of possible systemic toxicity.
